# Outcomes of COVID-19 patients intubated after failure of non-invasive ventilation: a multicenter observational study

**DOI:** 10.1038/s41598-021-96762-1

**Published:** 2021-09-06

**Authors:** Annalisa Boscolo, Laura Pasin, Nicolò Sella, Chiara Pretto, Martina Tocco, Enrico Tamburini, Paolo Rosi, Enrico Polati, Katia Donadello, Leonardo Gottin, Andrea Vianello, Giovanni Landoni, Paolo Navalesi, FERS, for the COVID-19 VENETO ICU Network, Ilaria Valeri, Giulio Andreatta, Leonardo Gandolfi, Alessandra Gadaldi, Nicolò Brumana, Edoardo Forin, Christelle Correale, Davide Fregolent, Pier Francesco Pirelli, Davide Marchesin, Matteo Perona, Nicola Franchetti, Michele Della Paolera, Caterina Simoni, Tatiana Falcioni, Alessandra Tresin, Chiara Schiavolin, Aldo Schiavi, Sonila Vathi, Daria Sartori, Alice Sorgato, Elisa Pistollato, Federico Linassi, Gian Lorenzo Golino, Laura Frigo, Eugenio Serra, Demetrio Pittarello, Ivo Tiberio, Ottavia Bond, Elisa Michieletto, Luisa Muraro, Arianna Peralta, Paolo Persona, Enrico Petranzan, Francesco Zarantonello, Tommaso Pettenuzzo, Alessandro Graziano, Alessandro De Cassai, Lorenzo Bernardi, Roberto Pianon, Flavio Badii, Enrico Bosco, Moreno Agostini, Paride Trevisiol, Antonio Farnia, Mario Peta, Lorella Altafini, Mauro Antonio Calò, Marco Meggiolaro, Francesco Lazzari, Ivan Martinello, Giorgio Fullin, Francesco Papaccio, Fabio Toffoletto, Alfeo Bonato, Camilla Sgarabotto, Fabio Baratto, Francesco Montacciani, Alessandra Parnigotto, Giuseppe Gagliardi, Ferraro Gioconda, Luigi Ongaro, Marco Baiocchi, Vinicio Danzi, Silvia De Rosa, Enrico Polati, Katia Donadello, Leonardo Gottin, Paolo Zanatta, Ezio Sinigaglia, Alessandra da Ros, Simonetta Marchiotto, Silvia Bassanini, Massimo Zamperini, Ivan Daroui, Walter Mosaner, Rosalba Lembo

**Affiliations:** 1 Institute of Anaesthesia and Intensive Care, Padua University Hospital, Padua, Italy; 2 Department of Medicine (DIMED), University of Padua, Via Vincenzo Gallucci 13, 35121 Padua, Italy; 3 Emergency Medical Services, Regional Department, AULSS 3, Venice, Italy; 4 Anaesthesia and Intensive Care Unit B, Department of Surgery, Dentistry, Gynaecology and Pediatrics, University of Verona, AOUI - University Hospital Integrated Trust, Verona, Italy; 5 Respiratory Pathophysiology Division, Department of Cardio-Thoracic, Vascular Sciences and Public Health, Padua University Hospital, Padua, Italy; 6 IRCCS San Raffaele Institute, Vita-Salute San Raffaele University, Milan, Italy; 7 U.O.C. Istituto di Anestesia e Rianimazione, Padua, Italy; 8 Azienda Ospedaliera-Università di Padova, Padua, PD Italy; 9 U.O.C. Anestesia e Rianimazione, Presidio Ospedaliero “San Martino” (AULSS 1 Dolomiti), Belluno, BL Italy; 10 U.O.C. Anestesia e Rianimazione, Ospedale di Vittorio Veneto (AULSS 2 Marca Trevigiana), Vittorio Veneto, TV Italy; 11 U.O.C. Anestesia e Rianimazione, Ospedale di Conegliano (AULSS 2 Marca Trevigiana), Conegliano, TV Italy; 12 U.O.C. Anestesia e Rianimazione, Ospedale di Montebelluna (AULSS 2 Marca Trevigiana), Montebelluna, TV Italy; 13 U.O.C. Anestesia e Rianimazione, Ospedale di Oderzo (AULSS 2 Marca Trevigiana), Oderzo, TV Italy; 14 U.O.C. Anestesia e Rianimazione, Ospedale Ca’ Foncello (AULSS 2 Marca Trevigiana), Treviso, Italy; 15 U.O.C. Anestesia, Rianimazione e Terapia Antalgica, Presidio Ospedaliero di Dolo (AULSS 3 Serenissima), Dolo, VE Italy; 16 U.O.C. Anestesia, Rianimazione e Terapia Antalgica, Presidio Ospedaliero di Mirano (AULSS 3 Serenissima), Mirano, VE Italy; 17 U.O.C. Anestesia e Rianimazione, Ospedale SS. Giovanni e Paolo (AULSS 3 Serenissima), Venezia, Italy; 18 U.O.C. Anestesia e Rianimazione, Ospedale dell’Angelo (AULSS 3 Serenissima), Mestre, VE Italy; 19 U.O.C. Anestesia e Rianimazione, Ospedali di San Donà di Piave e Jesolo (AULSS Veneto Orientale), San Donà di Piave, VE Italy; 20 U.O.C. Anestesia e Rianimazione, Ospedale di Cittadella (AULSS 6 Euganea), Cittadella, PD Italy; 21 U.O.C. Anestesia e Rianimazione, Ospedali Riuniti Padova Sud (AULSS 6 Euganea), Monselice, PD Italy; 22 U.O.C. Anestesia e Rianimazione, Ospedali di Rovigo e Trecenta (AULSS 5 Polesana), Rovigo, Italy; 23 U.O.C. Anestesia e Rianimazione, Ospedale Alto Vicentino (AULSS 7 Pedemontana), Santorso, VI Italy; 24 U.O.C. Anestesia e Rianimazione, Ospedale San Bassiano (AULSS 7 Pedemontana), Bassano del Grappa, VI Italy; 25 U.O.C Anestesia e Rianimazione, Ospedale di Vicenza (AULSS 8 Berica), Vicenza, VI Italy; 26 U.O. Anestesia e Rianimazione B, Azienda Ospedaliera Universitaria Integrata Verona, Verona, VR Italy; 27 U.O. Anestesia e Rianimazione A, Azienda Ospedaliera Universitaria Integrata Verona, Verona, VR Italy; 28 U.O.C Anestesia e Rianimazione, Ospedale Mater Salutis Di Legnago (AULSS 9 Scaligera), Legnago, VR Italy; 29 U.O.C Anestesia e Rianimazione, Ospedale Magalini di Villafranca (AULSS 9 Scaligera), Legnago, VR Italy; 30 Dipartimento di Anestesia, Rianimazione e Terapia Antalgica, IRCCS Sacro Cuore-Don Calabria, Negrar, VR Italy; 31 U.O.S. Terapia Intensiva, Dipartimento di Anestesia, Rianimazione e Terapia Antalgica, IRCCS Sacro Cuore-Don Calabria, Negrar, VR Italy; 32 U.O. Terapia Intensiva, Ospedale P. Pederzoli – Casa di Cura Privata SpA, Peschiera Sul Garda, VR Italy; 33 IRCCS San Raffaele Scientific Institute, Milan, MI Italy

**Keywords:** Respiratory distress syndrome, SARS-CoV-2

## Abstract

The efficacy of non-invasive ventilation (NIV) in acute respiratory failure secondary to SARS-CoV-2 infection remains controversial. Current literature mainly examined efficacy, safety and potential predictors of NIV failure provided out of the intensive care unit (ICU). On the contrary, the outcomes of ICU patients, intubated after NIV failure, remain to be explored. The aims of the present study are: (1) investigating in-hospital mortality in coronavirus disease 2019 (COVID-19) ICU patients receiving endotracheal intubation after NIV failure and (2) assessing whether the length of NIV application affects patient survival. This observational multicenter study included all consecutive COVID-19 adult patients, admitted into the twenty-five ICUs of the COVID-19 VENETO ICU network (February–April 2020), who underwent endotracheal intubation after NIV failure. Among the 704 patients admitted to ICU during the study period, 280 (40%) presented the inclusion criteria and were enrolled. The median age was 69 [60–76] years; 219 patients (78%) were male. In-hospital mortality was 43%. Only the length of NIV application before ICU admission (OR 2.03 (95% CI 1.06–4.98), *p* = 0.03) and age (OR 1.18 (95% CI 1.04–1.33), *p* < 0.01) were identified as independent risk factors of in-hospital mortality; whilst the length of NIV after ICU admission did not affect patient outcome. In-hospital mortality of ICU patients intubated after NIV failure was 43%. Days on NIV before ICU admission and age were assessed to be potential risk factors of greater in-hospital mortality.

## Introduction

The efficacy of non-invasive ventilation (NIV), including both Biphasic Positive Airway Pressure (BiPAP) and non-invasive Continuous Positive Airway Pressure (CPAP), in patients with acute respiratory failure (ARF) secondary to coronavirus disease 2019 (COVID-19) is still debated^1,2^.

On the one hand, some authors believe that NIV represents a questionable option and controlled mechanical ventilation should be established as soon as possible because of the risks of patient self-inflicted lung injury and delayed intubation^3^. On the other hand, solid evidence in favor of early intubation in COVID-19 ARF is still lacking, as several investigations failed to reveal a significant difference in mortality according to the time of intubation^4,5^.

Recent studies showed that a short NIV trial could be beneficial to treat COVID-19 mild-to-moderate hypoxemic ARF^6–14^. These investigations, however, were focused on the efficacy, safety and predictors of NIV failure applied outside the ICU^15–23^. Few studies reported the rate of NIV application in ICU, ranging from 11 to 50%, but the outcomes of critically ill patients, intubated after NIV failure, remain to be explored^6–9^.

Therefore, we designed this study aiming to investigate the incidence of in-hospital mortality in ICU patients receiving endotracheal intubation after NIV failure and to ascertain whether the length of NIV application before intubation may affect patient survival.

## Methods

The protocol was approved by the Institutional Ethical Committee of each participating centre (Ref: 4853AO20). The study was conducted in accordance with the Helsinki declaration and national regulation on study involving humans. Informed consent was obtained for each patient in compliance with national regulation and the recommendations of the Institutional Ethical Committee of Padova University Hospital.

We screened the records of all adult patients with confirmed SARS-CoV-2 infection, admitted into the twenty-five ICUs belonging to the COVID-19 VENETO ICU network^12^, between February 28 and April 28, 2020. We deemed eligible for analysis only patients who received endotracheal intubation after experiencing NIV (either CPAP or BiPAP) failure^12^. Patients exclusively receiving conventional and/or high-flow oxygen therapy or NIV, intubated after high-flow oxygen therapy, experiencing invasive mechanical ventilation without previous non-invasive treatments, with incomplete records or defined ‘do not intubate’ were excluded. Details on NIV setting, hospital organization and criteria for intubation are described in the supplementary material (Additional file, Methods).

The diagnosis of COVID-19 was made according to the WHO interim guidance (http://www.who.int/docs/default-source/coronaviruse/clinical-management-of-novel-cov.pdf). Laboratory confirmation of SARS-CoV-2 was defined as a positive result of real-time reverse transcriptase–polymerase chain reaction assay of nasopharyngeal swabs.

The following variables were collected: i) demographic data (age, gender, body mass index (BMI), onset of symptoms); ii) medical history (chronic diseases and long-term therapies, Charlson comorbidity index unadjusted for age^24^); iii) laboratory findings at ICU admission (blood count with formula, coagulation tests, C-reactive protein (CRP), procalcitonin, coagulation tests) and in-hospital treatments (i.e., ongoing therapies, including antiviral drugs and corticosteroids); iv) sequential organ failure assessment (SOFA) score at ICU admission; v) respiratory parameters before endotracheal intubation, i.e., positive end-expiratory pressure (PEEP), inspiratory pressure support above PEEP, fraction of inspired oxygen (FiO_2_), pH, arterial partial pressure of oxygen (PaO_2_), PaO_2_/FiO_2_, arterial partial pressure of carbon dioxide (PaCO_2_) and respiratory rate; vi) length of NIV application, either overall, before and after ICU admission; vii) the hospital location where NIV was applied, i.e., when NIV was applied exclusively in medical wards, respiratory high dependency units or emergency departments (ED), patients were included in the ‘out-of-ICU’ group. When NIV was applied exclusively after ICU admission patients were included in the ‘in-ICU’ group. When NIV was applied before and after ICU admission, patients were included in the ‘out- and in-ICU’ group; viii) complications occurred during the ICU stay (see full description listed in the additional file, Table 1); ix) ICU and hospital lengths of stay; x) hospital location before ICU admission (medical wards, respiratory high dependency units or ED); xi) hospital mortality.

For patients being readmitted or moved to a different hospital, only data from the first admission were considered. This study followed the ‘Strengthening the Reporting of Observational Studies in Epidemiology (STROBE) statement guidelines for observational cohort studies’^25^ (Additional files, Table 2). Each investigator had a personal username and password and entered data into a pre-designed online data acquisition system (www.covid19veneto.it). Patients’ privacy was protected by assigning a de-identified patient code. Prior to data analysis, two independent investigators and a statistician screened the database for errors against standardized ranges and contacted local investigators with any queries. Validated or corrected data were then entered into the database for final analysis.

### Statistical analysis

Statistical analysis was conducted using Stata 16 (Stata Statistical Software: Release 16.1 College Station, Texas USA: StataCorp) and R version 3.5.2.

Categorical data were presented as absolute numbers and percentages; for continuous data, normality was tested by Skewness and Kurtosis tests. Means and standard deviations were used when the variables were normally distributed, while medians and interquartile ranges were used in case of non-normally distributed variables. No imputation for missing data was planned.

Univariate analysis was used to investigate any difference between in-hospital survivors vs. non-survivors, concerning clinical characteristics, respiratory parameters before endotracheal intubation and the length of NIV application, both overall, before and after ICU admission.

Then, the independent predictors of in-hospital mortality have been identified through a stepwise multivariable regression model. This approach combines forward and backward selection methods in an iterative procedure (with a significance level of 0.05 both for entry and retention) to select predictors in the final multivariable model^26^. Independent variables used in the stepwise approach, and selected considering their clinical relevance, were age, Charlson comorbidity index, SOFA score at ICU admission, PaO_2_/FiO_2_, length of NIV application before, after ICU admission and the overall length of NIV.

Data were expressed as odds ratio (OR) and 95% confidence interval (95% CI).

Curves of cumulative incidence of in-hospital mortality were drawn to describe in-hospital mortality stratified by: i) patients’ characteristics (age); ii) length of NIV application prior to intubation; iii) and hospital location initially providing NIV. The median age and median length of NIV application, prior to intubation, of non-survivors were used as cut-off values for stratifying patients in two groups, as previously done^16^. Since discharge must be considered an ‘informative’ censoring^27^, cumulative incidence was calculated using methods accounting for competing risks and conventionally reported at 60-days. The Gray’s test was used to assess the difference between cumulative incidence functions. The observation period started at the day of endotracheal intubation. All statistical tests were 2-tailed, and statistical significance was defined as *p* < 0.05.

### Ethical approval

This was a multicenter, observational study performed in twenty-five hospitals of Veneto Region, Northern Italy, listed on the Acknowledgements. All the participating centers obtained Ethics Committee approval for the present research project, initially approved by the Institutional Ethical Committee of Padova University hospital on the 21st April, 2020 (Ref: 4853AO20). Local investigators were responsible for ensuring data integrity and validity. The study was conducted in accordance with the Helsinki declaration and national regulation on study involving humans. Informed consent was obtained for each patient in compliance with national regulation and the recommendations of the Institutional Ethical Committee of Padova University Hospital.

### Consent for publication

Informed consent was obtained for each patient in compliance with national regulation and the recommendations of the Institutional Ethical Committee of Padova University Hospital.

## Results

**Figure 1:**
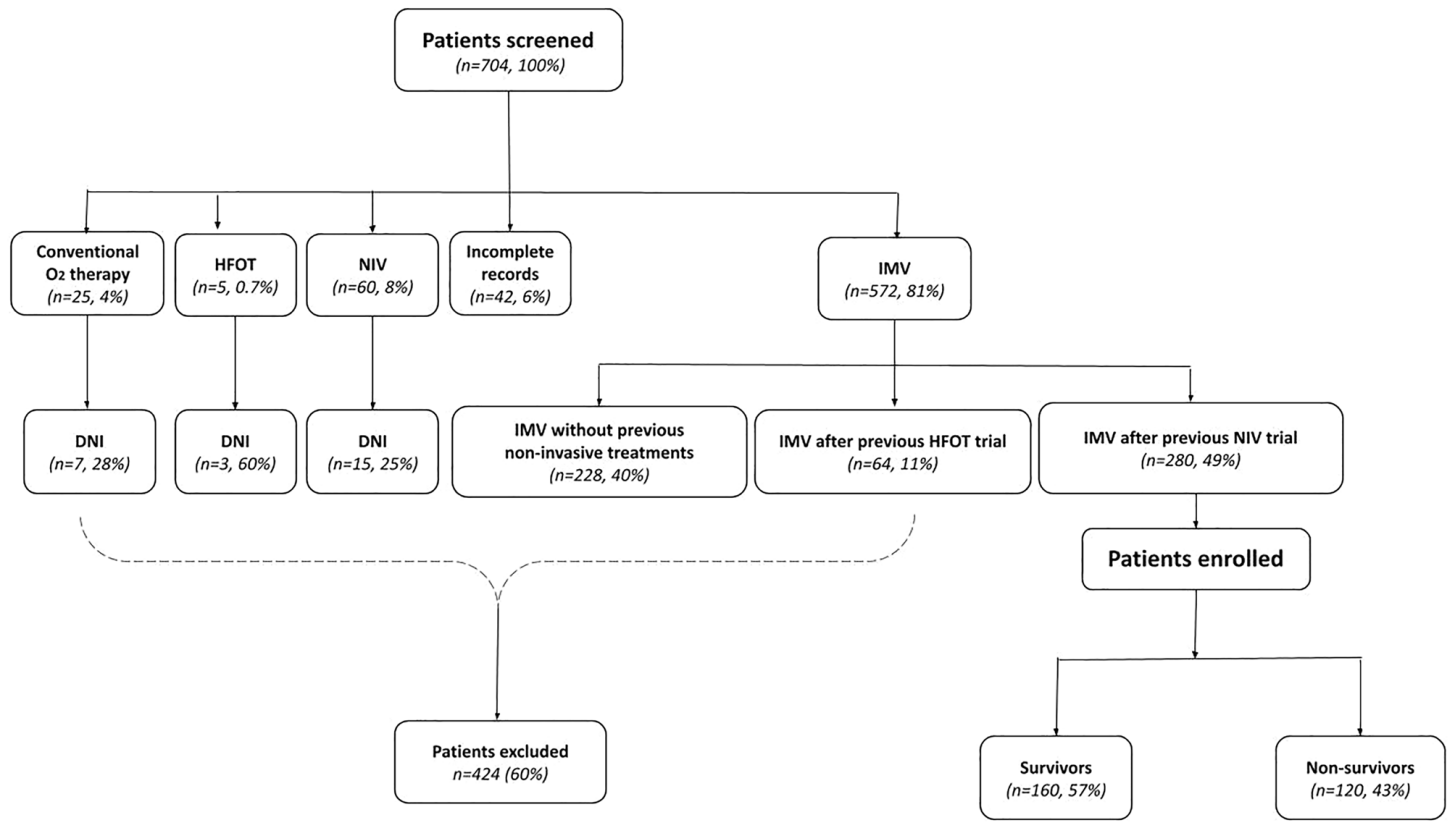
Flow chart of enrolled patients. HFOT: high flow oxygen therapy; NIV: non-invasive ventilation; IMV: invasive mechanical ventilation; DNI: ‘do not intubate’.

Data prospectively collected from a total of 704 consecutive patients with confirmed SARS-CoV-2 infection, admitted into the twenty-five ICUs belonging to COVID-19 VENETO ICU Network from February 28 to April 28, 2020^12^, were screened for inclusion criteria. Among them, 424 patients (60%) were excluded, while 280 (40%) were finally enrolled (Fig. 1).

Baseline demographic and clinical characteristics of the study population are presented in Table 1 or listed in the Additional files, Table 1.

**Table 1:** Description of clinical characteristics and respiratory parameters based on in-hospital mortality.

	Overall population, n = 280	in-hospital survivors n = 160 (57%)	in-hospital non-survivors n = 120 (43%)	OR of in-hospital mortality (95% CI)	*p* value
**Clinical characteristics**					
Age (years)	69 [60–76]	65 [57- 72]	73 [68–78]	0.98 (0.93—1.03)	0.50
Gender (male)	219 (78%)	122 (76%)	97 (81%)	1.31 (0.73—2.35)	0.36
BMI (kg/m^2^)	27 [24–30]	28 [25–31]	27 [25–30]	0.98 (0.93—1.03)	0.50
Charlson comorbidity index	**1 **[1–3]	**1 **[1, 2]	**2 **[1–4]	**1.22 (1.09—1.38)**	** < 0.01**
SOFA score at ICU admission	**5 **[4–8]	**4 **[3–7]	**6 **[4–10]	**1.21 (1.11—1.31)**	** < 0.01**
Onset of symptoms (days)	6 [3–9]	7 [3–9]	6 [3–10]	1.01 (0.96—1.05)	0.83
Hospitalization before ICU admission (days)	3 [1–5]	3 [1–4]	3 [1–7]	1.02 (0.99—1.06)	0.20
**Respiratory parameters before IMV**					
PEEP (cmH_2_O)	10 [8–10]	10 [5–16]	10 [5–16]	1.09 (0.93—1.28)	0.30
FiO_2_	**0.80 [0.60–1.00]**	**0.70 [0.40–1.00]**	**0.80 [0.21–1.00]**	**1.02 (1.01—1.04)**	** < 0.01**
PaO_2_/FiO_2_	**107 [77–150]**	**118 [90–175]**	**91 [73–131]**	**0.99 (0.98—0.99)**	** < 0.01**
PaCO_2_ (mmHg)	**40 [35–50]**	**39 [35–47]**	**43 [38–55]**	**1.04 (1.02—1.10)**	** < 0.01**
Respiratory rate (breaths/min)	20 [16–2780]	22 [16–25]	20 [16–28]	1.01 (0.97—1.05)	0.76
**Length of NIV application**					
Length of NIV before ICU admission (days)	1 [1–3]	**1 **[1, 2]	**2 **[1–4]	**1.18 (1.02–1.37)**	**0.03**
Length of NIV after ICU admission (days)	2 [1–3]	2 [1–3]	2 [1–4]	1.05 (0.92–1.20)	0.48
Overall length of NIV (days)	2 [1–4]	2 [1–3]	2 [1–5]	1.08 (0.99–1.18)	0.06

Data are expressed as median and InterQuartile Range [IQR] or number (%), Odds Ratios (OR) and 95% Confidence Interval (CI).

Bold values are statistically significant.

BMI: body mass index; SOFA: sequential organ failure assessment; ICU: intensive care unit; PEEP: positive end-expiratory pressure; PaO_2_/FiO_2_: ratio between partial pressure of arterial oxygen and fraction of inspired oxygen; PaCO_2_: partial pressure of carbon dioxide; NIV: non-invasive ventilation; IMV: invasive mechanical ventilation.

One-hundred-twenty patients (43%) died during the hospital stay. These patients showed an increased number of comorbidities (Charlson comorbidity index 2 [1–4] vs 1 [1, 2], *p* < 0.01), greater SOFA score at ICU admission (6 [4–10] vs 4 [3–7], *p* < 0.01) and more deteriorated gas exchange prior to endotracheal intubation (Table 1).

With respect to the hospital location initially providing NIV, 142 patients (51%) were exclusively treated ‘out-of-ICU’. Among those, 76 (54%) died before hospital discharge. A total of 82 patients (29%) received NIV only after ICU admission and 21 (36%) died. Finally, 56 patients (20%) failed ‘out-of and in-ICU’ NIV and 23 of them (41%) died. Worth mentioning, 147 (53%) patients received NIV before ICU admission in medical wards, while 77 (27%) in respiratory high dependency units, according to illness severity. Finally, 56 (20%) patients were directly admitted to ICU.

At univariate analysis, Charlson comorbidity index, SOFA score at ICU admission, FiO_2_, PaO_2_/FiO_2_, PaCO_2_ and the length of NIV before ICU admission were significantly related to in-hospital mortality (Table 1). On the contrary, at the multivariable logistic regression model, only age and the length of NIV before ICU admission were confirmed as independent predictors of in-hospital mortality (Table 2).

**Table 2:** Multivariable logistic regression analysis on the association between length of NIV application and in-hospital mortality.

	OR of in-hospital mortality (95% CI)	*p* value*
Age (years)	**1.18 (1.04–1.33)**	** < 0.01**
Charlson comorbidity index	0.96 (0.70–1.31)	0.80
SOFA score at ICU admission	1.19 (0.86–1.63)	0.29
PaO_2_/FiO_2_ before IMV	1.01(0.95–1.03)	0.55
Length of NIV before ICU admission (days)	**2.30 (1.06–4.98)**	**0.03**
Length of NIV after ICU admission (days)	1.16 (0.77–1.73)	0.48
Overall length of NIV (days)	1.12(0.77–1.64)	0.55

Data are expressed as Odds ratios (OR) and 95% Confidence Interval (CI).

Bold values are statistically significant.

*: Stepwise regression models, which combine forward and backward selection methods in an iterative procedure (with a significance level of 0.05 both for entry and retention) to select predictors in the final multivariable model. Independent variables used in the stepwise approach were selected considering their clinical relevance.

SOFA: sequential organ failure assessment; ICU: intensive care unit; PaO_2_/FiO_2_: ratio between partial pressure of arterial oxygen and fraction of inspired oxygen; NIV: non-invasive ventilation; IMV: invasive mechanical ventilation.

**Figure 2:**
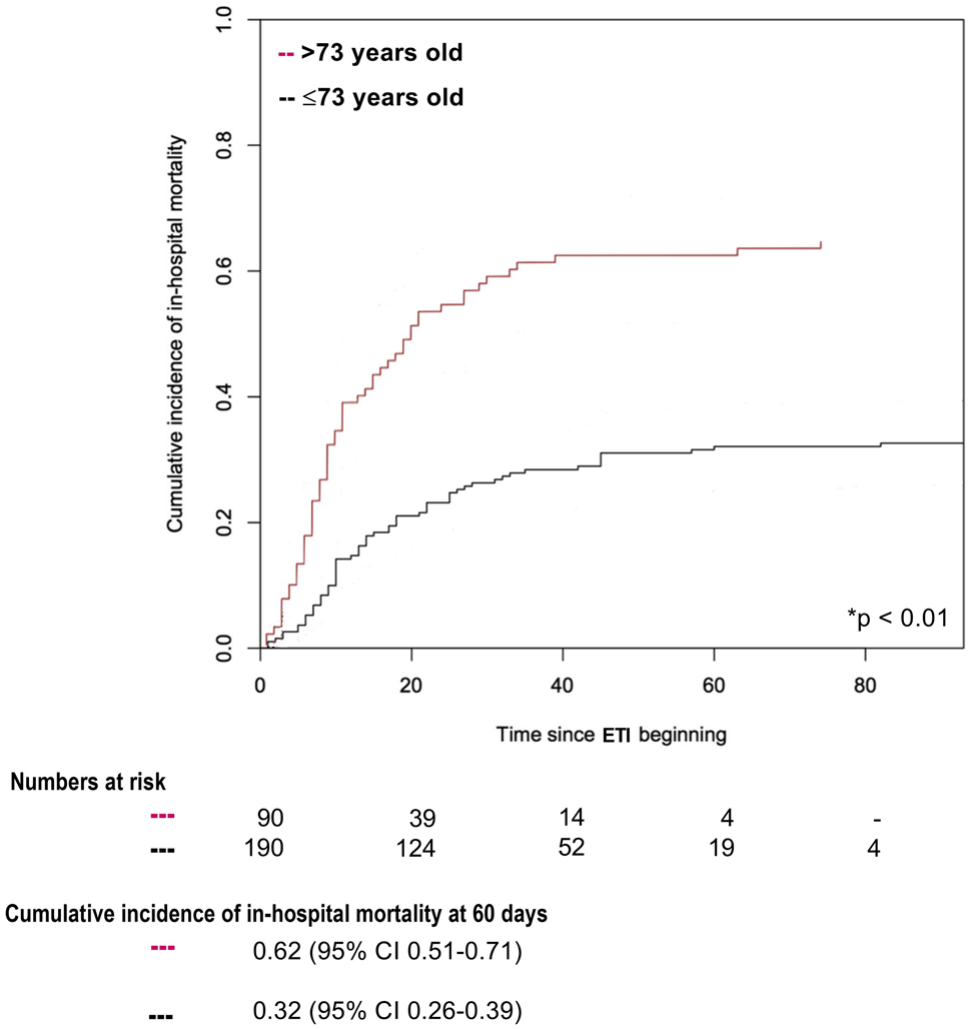
In-hospital mortality stratified by age (≤ or > 73 years). *p* Value Gray’s test was used for calculating equality of cumulative incidence function. The median age of non-survivors (= 73 years) was considered as the cut-off value for stratifying patients in two groups. NIV: non-invasive ventilation; ETI: endotracheal intubation.

**Figure 3:**
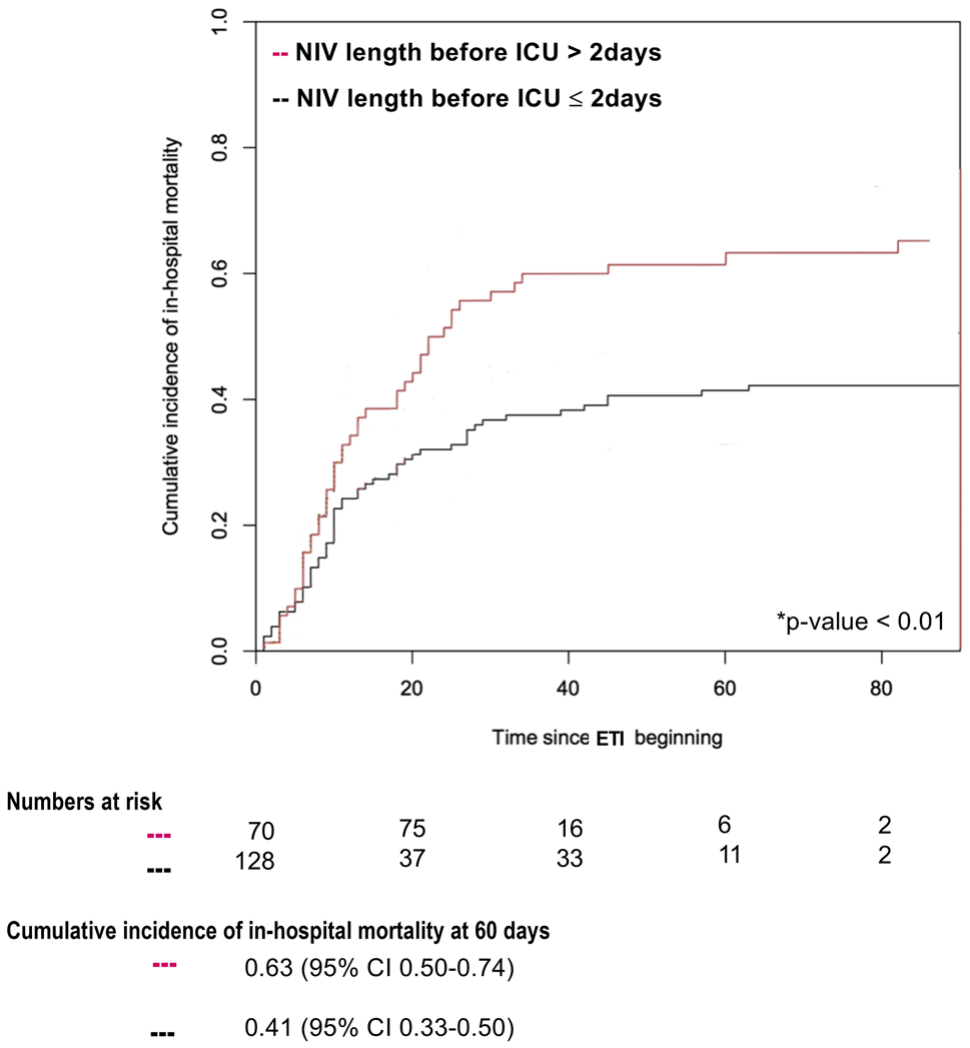
In-hospital mortality stratified by length of NIV application before ICU admission (≤ or > 2 days). *p* Value Gray’s test was used for calculating equality of cumulative incidence function. The median length of NIV application before ICU admission of non-survivors (= 2 days) was considered as the cut-off value for stratifying patients in two groups. NIV: non-invasive ventilation; ICU: intensive care unit; ETI: endotracheal intubation.

In the overall study population, patients older than 73 years (median age of non-survivors) showed an in-hospital mortality of 62% (95% CI 0.51–0.71), as opposed to patients ≤ 73 years (32%, 95% CI 0.26–0.39) (*p* < 0.01) (Fig. 2). Additionally, in-hospital mortality was significantly increased in patients receiving NIV for more than 2 days (median length of NIV application of non-survivors), as compared to those treated for 2 days or less (63% vs 41%; *p* < 0.01) (Fig. 3).

**Figure 4:**
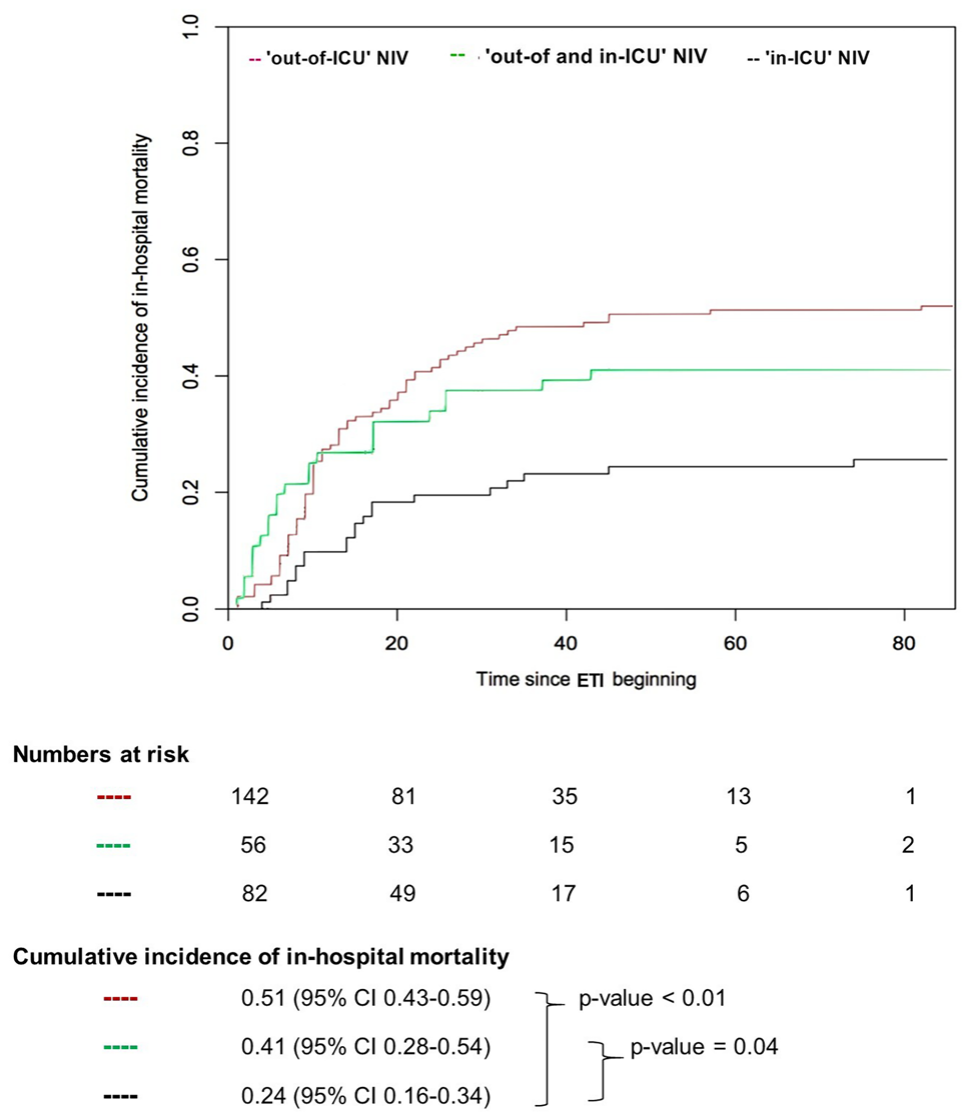
In-hospital mortality stratified by hospital location. *p* value Gray’s test was used for calculating equality of cumulative incidence function. When NIV was applied exclusively in medical wards, respiratory high dependency units or Emergency Department, patients were included in the ‘out-of-ICU’ group. When NIV was applied before and after ICU admission, patients were included in the ‘out- and in-ICU’ group. When NIV was applied exclusively after ICU admission patients were included in the ‘in-ICU’ group. NIV: non-invasive ventilation; ICU: intensive care unit; ETI: endotracheal intubation.

Finally, in-hospital mortality was higher in patients exclusively treated with ‘out-of-ICU’ NIV, as opposed to those exclusively treated with ‘in-ICU’ NIV (cumulative incidence 51% vs 24%, *p* < 0.01) or treated with NIV both outside and inside the ICU (cumulative incidence 51% vs 41%, *p* = 0.04) (Fig. 4).

## Discussion

This study, conducted during the first wave of COVID-19 pandemia, shows 43% in-hospital mortality among patients who underwent endotracheal intubation after NIV failure for SARS-CoV-2. Moreover, length of NIV application outside the ICU exceeding 48 h and age above 73 years were associated with greater mortality.

To the best of our knowledge, this is the first study focusing on the outcome of COVID-19 ICU patients intubated after NIV failure. Noteworthy, patients intubated after NIV failure showed a mortality rate no different from 292 patients receiving intubation without a previous NIV trial (42% vs 43%, *p* = 0.66) (Fig. 1), which suggests that attempting NIV did not worsen outcome even in case of intubation after failure.

Several previous studies described COVID-19 patients who underwent NIV outside ICU, often including patients receiving NIV as “ceiling” treatment^15,16,19,21–23,28^. Only a minority of these studies, however, reported the incidence of mortality of patients who were intubated after NIV failure. In keeping with our findings, Vaschetto et al. reported an in-hospital mortality of 41.0%, while Karagiannidis et al. reported a 30-day mortality of 49.6%^14,16^. Lower mortality rates were reported by Aliberti et al. and Franco et al. (26.5% and 26.7%, respectively)^20,23^. However, these two studies do not provide any information about patients’ clinical conditions at ICU admission, which makes any comparison with our results extremely problematic. The only relevant difference that can be noticed is the median age of the study population in the study by Aliberti et al. (60 [51–72] years)^20^, quite lower than ours (69 [60–76] years).

With respect to the length of NIV before tracheal intubation, our results are consistent with the findings of Vaschetto et al., describing a large population of COVID-19 patients treated with CPAP outside ICU^16^. In that study, 60-day in-hospital mortality was significantly higher in patients undergoing CPAP for more than 3 days (cumulative incidence 51%, 95% CI, 0.39–0.61) as compared to those receiving CPAP for 3 days or less (35%, 95% CI, 0.25–0.44)^16^.

While previous investigations were focused on the outcome of NIV delivered out of ICU^15,16,19,21–23,28^, our study provides detailed information on the outcome of intubation after NIV failure. Worth remarking, our data do not allow drawing any conclusion on the benefits of the application of NIV outside the ICU, as we do not consider the multitude of patients successfully treated with NIV in settings other than ICU in Veneto region during the study period^12^.

Our study presents some limitations. First of all, like many of the investigations on COVID-19, it is an observational study, thus it bears the limits of this study design. Second, in keeping with previous guidelines, we did not distinguish between patients treated with CPAP or BiPAP^1,29^, nor between patients supported with helmet or facial mask, nor between continuous or intermittent treatments. Therefore, our data do not allow to separately evaluate the benefits of BiPAP vs. CPAP or helmet vs. facial mask. Irrespective of the mode and interface, however, NIV guarantees maintenance of airway defence mechanisms and allows flexibility in applying and removing ventilatory assistance^30^. Third, NIV was mainly delivered through helmets, which made impossible measuring tidal volume^31^ and predicting the risk of patient self-inflicted lung injury^32^. Finally, it is worth remarking that the observed outcomes do not necessarily reflect those of patients treated outside a pandemic condition.

In conclusion, 43% of ICU patients receiving intubation after NIV failure died. Length of NIV before ICU admission and age were independent predictors of in-hospital mortality. Our findings suggest that prompt intubation is advisable in the case of lack of improvement after 2 days of NIV delivered outside ICU.

## Supplementary Information

Supplementary Information.

## Data Availability

The datasets used and analyzed during the current study are available from the corresponding author on reasonable request.

## Abbreviations

SARS-CoV-2: Severe acute respiratory syndrome coronavirus 2
NIV: Non-invasive ventilation
ARF: Acute respiratory failure
COVID-19: Coronavirus disease 2019
ICU: Intensive care unit
BiPAP: Biphasic positive airway pressure
CPAP: Continuous positive airway pressure
PaO_2_/FiO_2_: The ratio between arterial partial pressure of oxygen and inspired fraction of oxygen
BMI: Body mass index
CRP: C-reactive protein
SOFA: Sequential organ failure assessment
IMV: Invasive mechanical ventilation
PEEP: Positive end-expiratory pressure
FiO_2_: Fraction of inspired oxygen
PaO_2_: Arterial partial pressure of oxygen
PaCO_2_: Arterial partial pressure of carbon dioxide
CVVH: Continuous venous-venous hemofiltration
ED: Emergency department
OR: Odds ratio
CI: Confidence interval
ETI: Endotracheal intubation
