# Supplementary material for: Outcomes of COVID-19 patients intubated after failure of non-invasive ventilation: a multicenter observational study
Source: Sci Rep. 2021 Sep 6;11:17730. doi: 10.1038/s41598-021-96762-1 (PMC8421335; doi:10.1038/s41598-021-96762-1)
Supplement: Supplementary file 1 — Supplementary Information. [file 41598_2021_96762_MOESM1_ESM.docx]

**ADDITIONAL FILES**

**Outcomes of COVID-19 patients intubated after failure of non-invasive ventilation: a multicenter observational study.**

Annalisa Boscolo MD, Laura Pasin MD, Nicolò Sella MD, Chiara Pretto MD, Martina Tocco MD, Enrico Tamburini MD, Paolo Rosi MD, Enrico Polati MD, Katia Donadello MD, Leonardo Gottin MD, Andrea Vianello MD, Giovanni Landoni MD, Paolo Navalesi MD, FERS for the COVID-19 VENETO ICU Network.

**Methods. Criteria for intubation.**

**Table 1. Additional clinical characteristics of the study population.**

**Table 2. STROBE Statement—Checklist.**

**References.**

**METHODS.**

**NIV protocols.**

Continuous positive airway pressure (CPAP) was mainly delivered through helmets, via flow-meters (typically 40-60 l/min depending on the interface chosen) with a scale that allowed clinicians to separately set oxygen and air to regulate the inspiratory oxygen fraction (FiO2). Bilevel positive airway pressure (BiPAP) was most commonly delivered through helmets. Anti-bacterial and anti-viral filters were applied to the expiratory port of the circuit (1). The regional COVID-19 Network for coordination of SARS-CoV-2 outbreak in Veneto (Italy) shared specific protocols for NIV setting at the beginning of pandemic (2).

**Hospital organization.**

According to these regional guidelines, BiPAP was performed in the Respiratory High Dependency Units, while CPAP more often in the medical wards, to avoid in this less experienced setting the possible drawbacks of BiPAP related to a more problematic patient-ventilator interaction (2,3).

Nurse to patient ratios varied from a maximum of 1:4 to a minimum of 1:8, during days and nights in medical wards; while nurse to patient ratio was always 1:4 or 1:5 in respiratory high dependency units. Medical staff treating COVID-19 patients needing either CPAP or BiPAP was an ad-hoc mixed team, mainly internists, pneumologists, emergency physicians and cardiologists. NIV (either CPAP or BiPAP) was always supervisioned by anaesthesiologists actively working in ‘ad-hoc COVID-19 team’. Personnel was adequately trained for NIV; those who were not, received a short-organized training during pandemic based on video-tutorial (<https://www.youtube.com/channel/UCd2rd2RjAbwkqBVQnweE51Q/playlists>). Ward monitoring included SpO_2_, non-invasive blood pressure, ECG applied continuously or at a defined time point depending on the severity of the patient. Blood gas analysis was performed when clinically relevant. Additionally, invasive blood pressure measurement was available in respiratory high dependency units and emergency departments. During the first pandemic wave, Veneto never experienced unavailability of ICU beds (1) and all patients needing endotracheal intubation were promptly assisted in a dedicated ICU without delay.

**Criteria for intubation.**

Criteria for intubation were cardiac or respiratory arrest, inability to protect the airway, coma, severe hemodynamic instability associated to neurological impairment, intolerance to all non-invasive respiratory support interfaces, dyspnoea during non-invasive ventilation (NIV), respiratory rate >28 breaths/min and peripheral oxygen saturation (SpO2) below 92% after NIV attempt (1,2).

**Table 1. Additional clinical characteristics of the study population.**

|  | **Overall population,**  **n=280** | **in-hospital**  **survivors**  **n=160 (57%)** | **in-hospital**  **non-survivors**  **n=120 (43%)** | **p-value** |
| --- | --- | --- | --- | --- |
| ***Chronic diseases*** |  |  |  |  |
| ***COPD*** | 21 (7%) | 11 (6%) | 10 (8%) | 0.65 |
| ***Previous myocardial infarction*** | 19 (7%) | 10 (6%) | 19 (16%) | **0.01** |
| ***Congestive heart failure*** | 14 (5%) | 7 (4%) | 7 (6%) | 0.60 |
| ***Cognitive decline*** | 4 (1%) | 1 (1%) | 3 (3%) | 0.32 |
| ***Diabetes*** | 69 (25%) | 34 (21%) | 35 (29%) | 0.17 |
| ***Peripheral vascular disease*** | 36 (13%) | 9 (6%) | 27 (23%) | 0.90 |
| ***Moderate to severe CKD*** | 21 (7%) | 5 (3%) | 16 (13%) | **<0.01** |
| ***Long-term medical treatments*** |  |  |  |  |
| ***Ace inhibitors*** | 60 (21%) | 33 (21%) | 27 (22%) | 0.77 |
| ***Angiotensin II receptor antagonists*** | 45 (16%) | 15 (13%) | 30 (19%) | **<0.01** |
| ***Steroids*** | 20 (6%) | 8 (5%) | 12 (10%) | 0.16 |
| ***Statins*** | 55 (20%) | 28 (18%) | 27 (23%) | 0.36 |
| ***Immunosuppressants*** | 5 (2%) | 2 (1%) | 3 (3%) | 0.65 |
| ***In-hospital medical treatments*** |  |  |  |  |
| ***Antibiotic therapy*** | 102 (36%) | 54 (34%) | 48 (40%) | 0.32 |
| ***Prophylactic anticoagulants*** | 159 (57%) | 93 (58%) | 66 (55%) | 0.63 |
| ***Therapeutic anticoagulants*** | 85 (30%) | 36 (31%) | 49 (52%) | **<0.01** |
| ***Corticosteroids*** | 101 (36%) | 52 (36%) | 49 (46%) | 0.17 |
| ***Hydroxychloroquine/Lopinavir+Ritonavir*** | 184 (66%) | 108 (74%) | 76 (72%) | 0.53 |
| ***Laboratory findings at ICU admission*** |  |  |  |  |
| ***WBCs (×10^9^/L)*** | 9 [6-12] | 8 [6-12] | 9 [7-13] | **0.03** |
| ***CRP (mg/L)*** | 100 [22-197] | 100 [23-210] | 96 [21-170] | 0.54 |
| ***PCT (ng/ml)*** | 2 [0-5] | 1 [0- 4] | 2 [1-7] | 0.05 |
| ***Ferritin (ng/ml)*** | 1658 [766-2368] | 1422 [544-2295] | 1771 [972-2532] | 0.36 |
| ***IL-6 (ug/L)*** | 107 [64-250] | 76 [34-140] | 164 [94-605] | **<0.01** |
| ***PT (%)*** | 74 [64-95] | 76 [65-93] | 69 [64-100] | 0.89 |
| ***aPTT (sec)*** | 31 [26-34] | 30 [25-34] | 31 [28-34] | 0.05 |
| ***D-dimer (ng/ml)*** | 1079 [496-3917] | 734 [310-3011] | 2520 [877-6604] | **<0.01** |
| ***Fibrinogen (g/L)*** | 6 [4-7] | 6 [4-8] | 6 [4-7] | 0.64 |
| ***Additional parameters during NIV*** |  |  |  |  |
| ***Pressure support (cmH_2_O)*** | 12 [10-14] | 12 [8-14] | 12 [10-16] | 0.13 |
| ***pH*** | 7.41 [7.31-7.46] | 7.42 [7.35-7.47] | 7.37 [7.27-7.45] | **<0.01** |
| ***Clinical complications after ICU admission*** |  |  |  |  |
| ***Added respiratory infections*** | 113 (40%) | 56 (35%) | 57 (48%) | **0.04** |
| ***Acute kidney injury*** | 65 (23%) | 17 (11%) | 48 (40%) | **<0.01** |
| ***Kidney replacement therapy*** | 32 (11%) | 9 (6%) | 23 (19%) | **<0.01** |
| ***Acute hepatic injury*** | 29 (10%) | 10 (6%) | 19 (16%) | 0.43 |
| ***Myocardial injury*** | 3 (1%) | 1 (1%) | 2 (2%) | 0.99 |
| ***Vasoactive support*** | 187 (67%) | 97 (61%) | 90 (75%) | 0.01 |

Data are expressed as median and InterQuartile Range [IQR] or number (%), Odds ratios (OR) and 95% Confidence Interval (CI).

***Abbreviations:*** *COPD: chronic obstructive pulmonary disease; CKD: chronic kidney disease; WBC: white blood cells; CRP: C-reactive protein; PCT: procalcitonin; IL: interleukin; PT: prothrombin time; aPTT: activated partial thromboplastin time; ICU: intensive care unit.*

**Table 2. STROBE Statement—Checklist.**

|  | **Item No** | **Recommendation** | **Page No** |
| --- | --- | --- | --- |
| **Title and abstract** | 1 | (*a*) Indicate the study’s design with a commonly used term in the title or the abstract | 1-4 |
|  |  | (*b*) Provide in the abstract an informative and balanced summary of what was done and what was found | 1-4 |
| **Introduction** | | | |
| Background/rationale | 2 | Explain the scientific background and rationale for the investigation being reported | 5 |
| Objectives | 3 | State specific objectives, including any prespecified hypotheses | 6 |
| **Methods** | | | |
| Study design | 4 | Present key elements of study design early in the paper | 6 |
| Setting | 5 | Describe the setting, locations, and relevant dates, including periods of recruitment, exposure, follow-up, and data collection | 6 |
| Participants | 6 | (*a*) Give the eligibility criteria, and the sources and methods of selection of participants. Describe methods of follow-up | 6, Figure 1 |
|  |  | (*b*) For matched studies, give matching criteria and number of exposed and unexposed | - |
| Variables | 7 | Clearly define all outcomes, exposures, predictors, potential confounders, and effect modifiers. Give diagnostic criteria, if applicable | 6,7 |
| Data sources/ measurement | 8* | For each variable of interest, give sources of data and details of methods of assessment (measurement). Describe comparability of assessment methods if there is more than one group | - |
| Bias | 9 | Describe any efforts to address potential sources of bias | - |
| Study size | 10 | Explain how the study size was arrived at | Figure 1 |
| Quantitative variables | 11 | Explain how quantitative variables were handled in the analyses. If applicable, describe which groupings were chosen and why | 7 |
| Statistical methods | 12 | (*a*) Describe all statistical methods, including those used to control for confounding | 8 |
|  |  | (*b*) Describe any methods used to examine subgroups and interactions | 8 |
|  |  | (*c*) Explain how missing data were addressed | 8 |
|  |  | (*d*) If applicable, explain how loss to follow-up was addressed | 8 |
|  |  | (*e*) Describe any sensitivity analyses | - |
| **Results** | | |  |
| Participants | 13* | (a) Report numbers of individuals at each stage of study—eg numbers potentially eligible, examined for eligibility, confirmed eligible, included in the study, completing follow-up, and analysed | 9, Figure 1 |
|  |  | (b) Give reasons for non-participation at each stage | Figure 1 |
|  |  | (c) Consider use of a flow diagram | Figure 1 |
| Descriptive data | 14* | (a) Give characteristics of study participants (eg demographic, clinical, social) and information on exposures and potential confounders | 9 |
|  |  | (b) Indicate number of participants with missing data for each variable of interest | - |
|  |  | (c) Summarise follow-up time (eg, average and total amount) | 9 |
| Outcome data | 15* | Report numbers of outcome events or summary measures over time | 10 |
| Main results | 16 | (a) Give unadjusted estimates and, if applicable, confounder-adjusted estimates and their precision (eg, 95% confidence interval). Make clear which confounders were adjusted for and why they were included  (b) Report category boundaries when continuous variables were categorized  (c) If relevant, consider translating estimates of relative risk into absolute risk for a meaningful time period | 10 |
| Other analyses | 17 | Report other analyses done—eg analyses of subgroups and interactions, and sensitivity analyses | - |
| **Discussion** |  |  |  |
| Key results | 18 | Summarise key results with reference to study objectives | 11, 12 |
| Limitations | 19 | Discuss limitations of the study, taking into account sources of potential bias or imprecision.  Discuss both direction and magnitude of any potential bias | 12 |
| Interpretation | 20 | Give a cautious overall interpretation of results considering objectives, limitations, multiplicity of analyses, results from similar studies, and other relevant evidence | 12 |
| Generalisability | 21 | Discuss the generalisability (external validity) of the study results | 12 |
| **Other information** |  |  |  |
| Funding | 22 | Give the source of funding and the role of the funders for the present study and, if applicable, for the original study on which the present article is based | 14 |

**References.**

1. Vaschetto R, Barone-Adesi F, Racca F, Pissaia C, Maestrone C, Colombo D, et al. Outcomes of COVID-19 patients treated with continuous positive airway pressure outside ICU. ERJ Open Research 2021; doi:10.1183/23120541.00541-2020.
2. Pasin L, Sella N, Correale C, Boscolo A, Rosi P, Saia M, et al. Regional COVID-19 Network for Coordination of SARS-CoV-2 outbreak in Veneto, Italy. J Cardiothorac Vasc Anesth. 2020;34(9):2341-2345.
3. Costa R, Navalesi P, Spinazzola G, Ferrone G, Pellegrini A, Cavaliere F, et al. Influence of ventilator settings on patient-ventilator synchrony during pressure support ventilation with different interfaces. Intensive Care Med 2010;36(8):1363-70.
